# Case Report: A case of anti-mGluR1 antibody-associated encephalitis treated with efgartigimod combined with rituximab

**DOI:** 10.3389/fimmu.2026.1928007

**Published:** 2026-09-03

**Authors:** JunTong Liu, WenPing Zhu, Lan Gao, Chao Zhao

**Affiliations:** 1 Department of Neurology, Xi’an International Medical Center Hospital, Xi’an, Shaanxi, China; 2 Department of Neurology, Tangdu Hospital, The Fourth Military Medical University, Xi’an, Shaanxi, China

**Keywords:** anti-metabotropic glutamate receptor 1, autoimmune encephalitis, efgartigimod, rituximab, treatment

## Abstract

Anti-metabotropic glutamate receptor 1 (mGluR1) encephalitis is a rare autoimmune disorder affecting both the central and peripheral nervous systems, typically characterized by a subacute cerebellar syndrome. Its effective treatment remains undefined. Here, we present the case of a 16-year-old male student who experienced dizziness upon walking and standing. Symptoms gradually worsened, leading to psychiatric and behavioral abnormalities. Neurological examination revealed somnolence, dysarthria, a broad-based unsteady gait, a prominent head tremor, and limb ataxia. The first magnetic resonance imaging scan showed no obvious abnormality. The background rhythm on the electroencephalogram was normal, with no epileptiform discharges observed. A whole-body PET/CT scan revealed a mild reduction in glucose metabolism in the left frontal and temporal lobes. Lumbar puncture showed normal cerebrospinal fluid pressure but elevated white blood cell count and protein level. The diagnosis of anti-mGluR1 encephalitis was confirmed by positive anti-mGluR antibody testing, specifically showing an anti-mGluR1-IgG titer of 1:1000 in the cerebrospinal fluid and an anti-mGluR1-IgG1 titer of 10,000 in the serum. The patient did not respond to first-line therapy with corticosteroids. During disease progression, innovative sequential therapy with efgartigimod, an FcRn antagonist, was followed by the anti-CD20 monoclonal antibody rituximab (RTX) to reduce the risk of profound B-cell depletion. The patient exhibited significant clinical improvement, evidenced by a reduction in the Modified Rankin Scale (mRS) score from 4 to 3 and a decrease in the Scale for Assessment of Ataxia (SARA) score from 40 to 13. Efgartigimod effectively cleared pathogenic IgG, whereas RTX induced rapid B-cell depletion to prevent disease recurrence, thereby demonstrating synergistic efficacy. At the 12-month follow-up, the patient’s clinical symptoms continued to improve, with no observed relapse. This case report underscores the importance of early diagnosis and therapeutic intervention, highlighting the need to suspect this condition based on its characteristic clinical presentation.

## 1 Introduction

Anti-mGluR1 antibody-associated encephalitis is an uncommon autoimmune disorder mediated by IgG1 antibodies targeting the metabotropic glutamate receptor (1). It is characterized by immune activation against central nervous system antigens, such as cerebellar Purkinje cells, leading to acute or subacute encephalopathy (2). The typical clinical manifestations include cerebellar ataxia, cognitive impairment, and psychiatric symptoms, with additional symptoms potentially including head tremors, seizures, or involuntary movements (3, 4). First-line immunotherapy during the acute phase typically involves high-dose intravenous corticosteroids, intravenous immunoglobulin (IVIG), or plasma exchange (PE), administered either singly or in combination (5, 6). For refractory cases, second-line therapies such as rituximab or cyclophosphamide are recommended (6). Despite the potential efficacy of these therapeutic interventions, their effectiveness is often constrained by delayed onset and suboptimal response in certain patients (7, 8). In clinical practice, the implementation of these treatment regimens faces challenges, including insufficient clearance of antibodies from cerebrospinal fluid, high costs, invasiveness, supply shortages, coagulation disorders, and infection risks (5, 9, 10), all of which can impose a significant burden on patients. Consequently, there is a pressing need for the development of rapid, effective, and well-tolerated therapeutic strategies for anti-mGluR1 encephalitis to address clinical requirements.

Recent advancements have highlighted novel therapies targeting the neonatal Fc receptor (FcRn), demonstrating that inhibiting IgG-FcRn recycling can markedly expedite the degradation of pathogenic IgG, particularly the IgG1 subclass, without substantially impacting other immunoglobulin subclasses (11, 12). The FcRn antagonist efgartigimod has been validated in phase III trials (ADAPT study) for myasthenia gravis, where it improved functional scores and reduced relapse rates (13), and has shown preliminary efficacy in neuroimmunological disorders such as NMOSD (14)and anti-NMDAR encephalitis (15). Importantly, the primary pathogenic mechanism of anti-mGluR1 encephalitis involves neural cell dysfunction mediated by IgG1 antibodies, and the high selectivity of efgartigimod in clearing IgG1 positions it as a potentially optimized treatment option for this condition (3). In this context, we report a case of an adolescent with anti-mGluR1 encephalitis in whom combined immunotherapy with efgartigimod and sequential rituximab resulted in favorable outcomes.

## 2 Case description

### 2.1 Clinical course and diagnostic workup

A 16-year-old Han Chinese boy was admitted to our hospital due to “progressive dizziness persisting for ten days.” On November 11, 2024, the patient experienced a sudden onset of persistent dizziness of unknown etiology, accompanied by nausea, non-projectile vomiting, and gait instability. Notable fine motor deficits were observed, including difficulty grasping objects, accompanied by dysarthria. There was an absence of bilateral tinnitus or hearing loss, and the symptoms did not abate with rest. As the condition advanced, local treatments proved ineffective. Upon referral to our institution (on November 21, 2024), the patient presented with newly developed acute neuropsychiatric features, including agitation, paroxysmal vocalizations, disorganized cognition, drowsiness, and a vacant expression. The outpatient diagnosis was confirmed as autoimmune encephalitis. Throughout the illness, the patient exhibited mental lethargy and a decreased appetite, although weight remained stable and bowel and bladder functions were normal. There was no significant past medical, personal, or family history.

Physical examination upon admission revealed a body mass index (BMI) of 17.31 kg/m² (weight 53 kg, height 175 cm), with no significant abnormalities detected in other major systems. The neurological assessment revealed the following: the patient exhibited a drowsy state of consciousness and demonstrated poor cooperation, corresponding to a modified Rankin Scale (mRS) score of 4. Evaluation of cerebellar function indicated ataxia, with a Scale for the Assessment and Rating of Ataxia (SARA) score of 40/40, an inability to complete coordination tasks, and a positive Romberg test. Motor function assessment showed reduced muscle tone in the limbs, with muscle strength rated at 5/5. The examination of deep and superficial sensations was hindered by poor cooperation. Reflex testing revealed bilateral biceps and knee tendon reflexes were graded as (++), while pathological signs such as Hoffmann and Babinski were negative.

**Figure 1:**
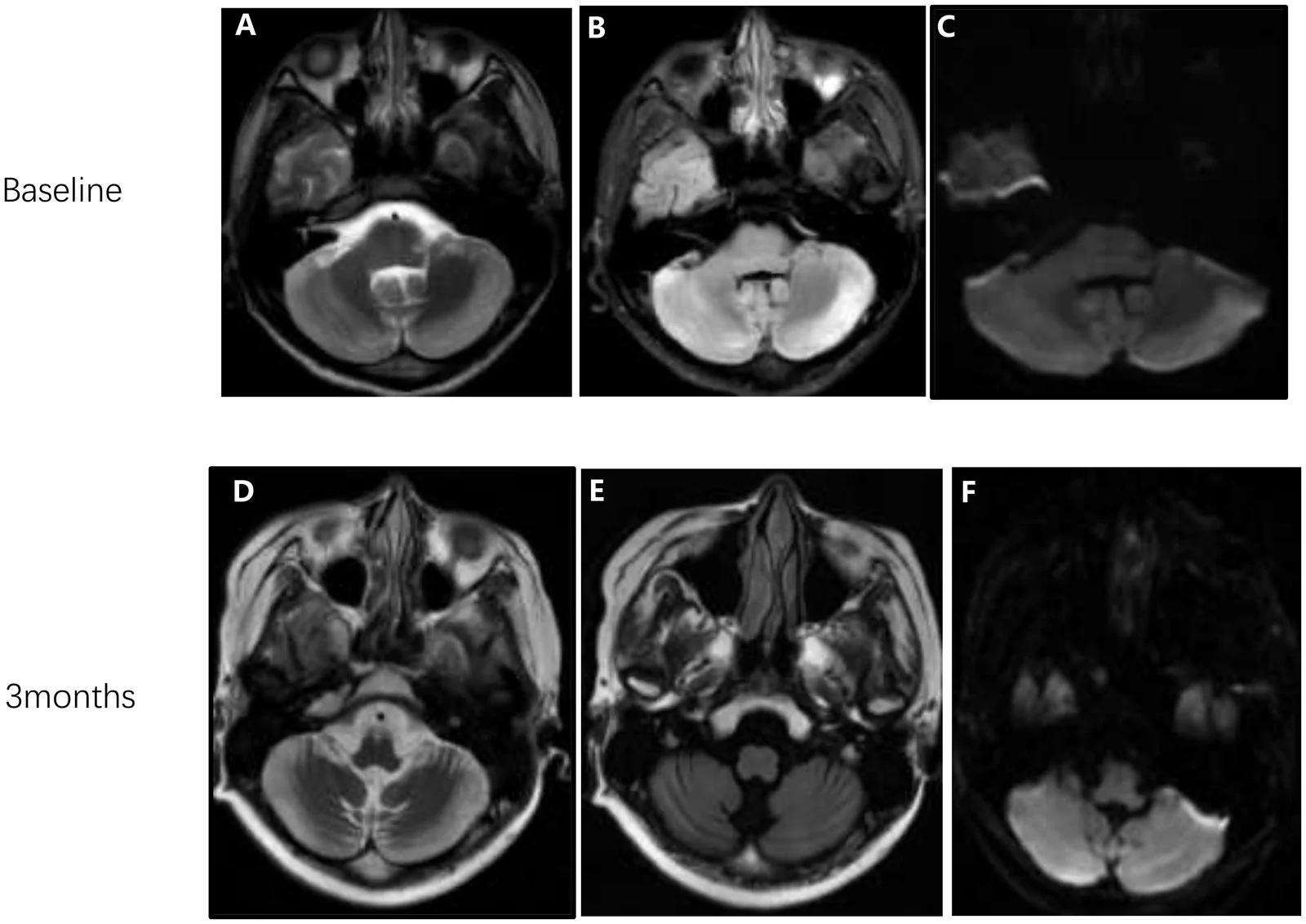
Magnetic resonance imaging (MRI) of the brain showed no abnormal signs. **(A–C)** T2WI, T2-FLAIR, and DWI axial views of the temporal lobe and cerebellum. T2WI, T2-weighted imaging; FLAIR, T2-weighted fluid-attenuated inversion recovery; DWI, diffusion-weighted imaging. **(D–F)** Showed mild bilateral cerebellar hemisphere atrophy.

Auxiliary examinations, including routine blood tests, assessments of liver and kidney function, electrolytes, tumor markers, autoimmune spectrum (ANA/ENA, etc.), thyroid function, and infection screenings (hepatitis B, syphilis, HIV), returned normal results. Imaging studies, including CT scans of the chest, abdomen, and pelvis, as well as testicular ultrasound, showed no evidence of tumors. Cranial MRI, incorporating DWI/MRA sequences, did not reveal any structural, vascular, or diffusion abnormalities (**Figures 1A–C**). Cerebrospinal fluid analysis demonstrated a white blood cell count of 84 × 10^6/L, predominantly lymphocytes (>70%), with normal glucose and chloride levels, mildly elevated CSF protein (600 mg/L; ref: 150–450 mg/L), and a negative Gram stain.

**Figure 2:**
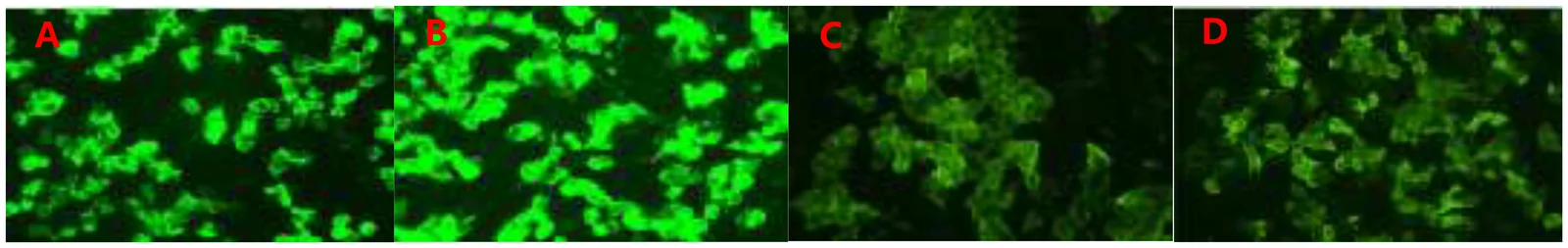
Metabotropic glutamate receptor 1 antibodies in serum and CSF validated by cell-based assays. mGluR1-IgG levels in serum [**(A)** 1:10000] and CSF [**(B)** 1:1000] at disease onset, and in serum [**(C)** 1:32] and CSF [**(D)** 1:10] over fifteen weeks after efgartigimod treatment initiation.

In light of the characteristics associated with cerebellar syndrome, initial serum and cerebrospinal fluid (CSF) tests for cerebellitis antibodies, including anti-NMDAR, AMPAR1/2, LGI1, CASPR2, GABABR, GAD65, as well as ganglioside antibodies GM1, GD1b, and GQ1b, yielded negative results. Subsequent screening for less common antibody types revealed a CSF anti-mGluR1-IgG titer of 1:1000 and a serum anti-mGluR1-IgG1 titer of 10,000. Cell-based assays (CBA) were used to detect antibodies in CSF and serum. A whole-body PET-CT scan, conducted to investigate potential occult tumors, showed mild hypometabolism in the left frontal and temporal lobes, without any findings indicative of malignancy. The routine electroencephalogram demonstrated a normal background rhythm and showed no epileptiform discharges. Based on the elevated anti-mGluR1 antibody titers (serum 1:10,000; CSF 1:1,000; **Figures 2A, B**) and the patient’s rapidly progressive cerebellar and neuropsychiatric symptoms, a diagnosis of anti-mGluR1 antibody-associated autoimmune encephalitis was established.

### 2.2 Clinical assessment of immunotherapy profiles

Outcome measures in studies of autoimmune encephalitis primarily relied on established, widely used tools such as the modified Rankin Scale (mRS). The mRS consists of six grades (0–5 points) and predominantly captures the impact of motor deficits on functional independence. However, the mRS was developed to assess stroke outcome, and it fails to detect clinically relevant long-term symptoms in these patients (30).

The Scale for Assessment and Rating of Ataxia (SARA) is well-recognized as a reliable instrument for the quantification of ataxia in children and adults. The SARA is an ataxia rating scale ranging from an ‘optimal’ score of 0 to a ‘most severe’ score of 40, comprising subscores for gait, kinetics, and speech. Its application in clinical settings is facilitated by its brevity and ease of administration (31).

### 2.3 Clinical course and therapeutic intervention

At admission, the patient’s modified Rankin Scale (mRS) score was 4, and the Scale for Assessment and Rating of Ataxia (SARA) score was 40. Intravenous methylprednisolone was administered (1000 mg once daily for 3 days as pulse therapy, then reduced to 500 mg once daily for 2 days), followed by oral methylprednisolone tablets (40 mg once daily) with gradual tapering (reduced by 4 mg every 2 weeks). The patient’s symptoms of dizziness, head shaking, and ataxia showed no significant improvement. On day 12 of admission, the first round of efgartigimod treatment was initiated (10mg/kg once weekly via IV infusion for 4 weeks). By the end of treatment (day 33 of admission), the patient’s slurred speech and ataxia had improved compared to baseline, but walking still required assistance (mRS: 3, SARA: 25).To further eliminate pathogenic B cells and prevent disease recurrence, low-dose rituximab (100mg once weekly via IV infusion for 3 weeks) was administered. At discharge (January 16, 2025), the patient’s mRS was 3 and SARA was 23.

### 2.4 Follow-up

Post-discharge, he continued rehabilitation exercises with periodic follow-ups. One month later (February 16, 2025), during follow-up, mRS remained at 3 and SARA improved to 16, prompting initiation of the second round of efgartigimod treatment. By its completion, mRS decreased to 2 and SARA to 13 (**Table 1**).The patient achieved near-independence in daily activities, no longer required assistance while walking, exhibited significantly reduced slurred speech, and showed near-resolution of psychiatric symptoms. No drug allergies or adverse reactions occurred during treatment. Follow-up brain MRI (comparing images from November 27, 2024, and March 11, 2025) revealed mild bilateral cerebellar hemisphere atrophy (**Figures 1D–F**).Repeat lumbar puncture and serology examination showed a reduction in serum anti-mGluR1-IgG titers to 1:32 and CSF titers to 1:10 (**Figures 2C, D**). A follow-up period extending over one year revealed no relapses and a continued improvement in clinical symptoms. A timeline of the patient’s diagnostic and treatment process is provided (**Table 1**).

**Table 1:** Patient treatment and diagnostic timeline.

Time	2024-11-11	2024-11-21	1month	3months	After 6months		After 12months
Clinical manifestations	Dizziness,headache, Vomiting, nausea, ataxia, hypersomnia	Psychobehavioral abnormalities, irritability, severe functional dependency	Persistent dysarthria	Significant improvement in dysarthria; able to ambulate independently	Mild dysarthria and no new positive signs were found		The patient’s family reported that the symptoms do not impact daily life
Accessory examination	Brain MRI and EEG: No abnormal signs	CSF detection: elevated WBC count and protein level AE antibody: serum & CSF: mGluR1 antibody positive. CSF; IgG:12.1g/L.	IgG: 2.59g/L; CD20+B:6.59%	Brain MRI: mild bilateral cerebellar hemisphere atrophy; AE antibody: serum & CSF: mGluR1 antibody titer decreased; IgG: 3.91g/L. CD20+B:0.11%	Brain MRI: mild bilateral cerebellar hemisphere atrophy; IgG: 1.52g/L		
Evaluation Scales	mRS: 4;SARA:40		mRS: 3;SARA:25	mRS: 3;SARA:16	mRS: 2;SARA:13		mRS: 2;SARA:8
Treatment	Large-dose methylprednisolone sodium succinate pulse therapy: 1000mg for 3 consecutive days500mg for 2 consecutive days, and then oral prednisone acetate 40mg gradually reduced until discontinued; Efgartigimod (10mg/kg once a week for four weeks).		Efgartigimod immunotherapy has been administered for four weeks.	Low-dose rituximab (100mg once weekly via IV infusion for 3 weeks) has been administered	The second round of Efgartigimod treatment has been administered for four weeks	-	
Patient Perspective	The patient and their family have gone from a lack of understanding of the disease to providing positive feedback during monthly follow-ups.		For diseases related to the autoimmune system, the diagnosis and treatment process is prolonged, and symptoms may fluctuate during this period.				

Detailed clinical status was evaluated by a series of scales at baseline before treatment and continuously for 1 year after treatment. mRS, the modified Rankin Scale score; SARA, the Scale for the Assessment and Rating of Ataxia; IgG, Serum levels of Immunoglobulin G assay; CSF, Cerebrospinal fluid; WBC, White blood cell count; EEG, Electroencephalogram.

## 3 Discussion

The case reports a patient diagnosed with anti-mGluR1 encephalitis. In the case under discussion, the patient initially presented with symptoms of dizziness and unsteady gait, followed by manic and agitated psychiatric manifestations, which strongly suggested encephalitis. Based on neurological examination, the lesion is localized to the vestibular-cerebellar and cerebral cortex. Although early cranial MRI did not reveal any clear abnormalities, CSF analysis indicated a mild increase in white blood cell count and elevated protein levels. Differential diagnosis between autoimmune and infectious neurological disorders is required. The patient in this case did not present with high fever or meningeal signs, had only a mild elevation of white blood cells in the CSF, and showed no abnormalities on brain MRI. Both serum and CSF tests were positive for anti-mGluR1 antibodies. These findings cannot be explained by infectious encephalitis alone; therefore, tumor must be considered as a differential diagnosis. Arguments against a tumor include the acute onset of symptoms, absence of definite lesions on MRI, and a favorable response to immunotherapy.

Anti-mGluR1 encephalitis typically presents acutely or subacutely, predominantly affecting middle-aged individuals and rarely observed in pediatric populations (3, 16). A subset of patients may experience prodromal symptoms, such as infections, prior to the onset of neurological manifestations, which frequently present as cerebellar ataxia and may be accompanied by behavioral changes and cognitive impairments (3, 17). In pediatric patients, significant chorea and dystonia affecting the oral-facial and limb regions may manifest (16). Approximately 50% of these patients may exhibit normal cerebrospinal fluid (CSF) cell counts and initial magnetic resonance imaging (MRI) results (4, 18); however, over time, some patients may develop positive MRI findings (3).

This progression in MRI results is hypothesized to be associated with the degeneration of Purkinje cells due to continuous exposure to antibodies (4, 19).

As noted, this patient initially presented with dizziness and unsteady gait, followed by manic and agitated psychiatric manifestations suggestive of encephalitis. Although early cranial MRI did not reveal any clear abnormalities, CSF analysis indicated a mild increase in white blood cell count and elevated protein levels. Given the prominent cerebellar syndrome and despite negative results from initial common cerebellitis antibody screening, we expanded the testing to include rare antibodies, ultimately leading to a confirmed diagnosis.

Early initiation of immunotherapy in patients with autoimmune encephalitis is closely associated with functional recovery and reduced neurological damage (20, 21). Anti-mGluR1 antibodies, primarily of the IgG1 subclass, are considered pathogenic (3). There appears to be a correlation between antibody titers and the severity of clinical symptoms (3). Prolonged exposure to pathogenic antibodies may result in irreversible damage to central nervous system functions (1, 22). The patient exhibited severe clinical symptoms and elevated antibody titers in both serum and cerebrospinal fluid; consequently, a rapid and effective treatment strategy was required to address the clinical urgency.

Studies have demonstrated that the FcRn antagonist efgartigimod can block the binding of FcRn to IgG, leading to IgG degradation in lysosomes (11).Compared to traditional immunotherapy, FcRn antagonists offer the advantage of rapidly reducing antibody levels in the short term, alleviating clinical symptoms, improving prognosis, reducing relapse rates, and decreasing corticosteroid usage (23, 24). This demonstrates significant potential for application in patients with acute critical neuroimmunological conditions and has been extended for use in various autoimmune diseases (25–28).

In this case, the patient achieved rapid disease control following the administration of efgartigimod during hospitalization, with a swift reduction of total serum IgG levels, while levels of non-IgG immunoglobulins (IgA, IgM, IgD, IgE) were not significantly affected. We also observed that the decrease in antibody titers was significantly correlated with the improvement in the patient’s clinical symptoms and scale scores. Autoimmune encephalitis is an antibody-mediated disorder of the central nervous system (CNS), where a blood-brain barrier (BBB) exists between the CNS and the circulatory system. Therefore, in addition to eliminating pathogenic antibodies from the peripheral circulation, it is also necessary to effectively remove antibodies that have already entered the central nervous system. It is generally accepted that macromolecular drugs cannot cross the blood-brain barrier, and the clearance of antibodies already present in the central nervous system is usually limited. In this patient, it was observed that after efgartigimod therapy, the serum anti-mGluR1 antibody titer decreased from 1:10000 to 1:32, and CSF anti-mGluR1 antibody titer decreased from 1:1000 to 1:10. Pathogenic antibodies in both serum and CSF were significantly reduced, and total serum IgG levels were also reduced significantly. There is currently no direct evidence that efgartigimod can cross the blood-brain barrier in patients with encephalitis; however, based on the clinical effects and laboratory results observed in this case, efgartigimod appears to have effectively cleared anti-mGluR1 antibodies from both serum and the central nervous system.

Under inflammatory conditions, we hypothesize two potential mechanisms for efgartigimod-mediated antibody clearance within the CNS: first, reducing serum antibody concentrations to limit further CNS penetration; and second, directly modulating the CNS-peripheral antibody balance via action on abundant FcRn expressed on blood-brain barrier endothelial cells (29).

Although efgartigimod exerts therapeutic effects by rapidly clearing pathogenic antibodies, it does not directly inhibit antibody production. In light of these considerations, we employed a sequential low-dose rituximab regimen to achieve rapid B cell depletion. This combined therapeutic approach significantly alleviated symptoms and contributed to the long-term stabilization of the patient’s condition, with no notable adverse reactions recorded.

Currently, the FcRn antagonist efgartigimod is recognized for its considerable potential in the treatment of various IgG-mediated autoimmune diseases (23–25, 27). To our knowledge, this represents the first case report using efgartigimod in conjunction with sequential low-dose rituximab for the acute management of anti-mGluR1 encephalitis, yielding promising therapeutic outcomes. However, this study is subject to certain limitations: the observed efficacy during the patient’s hospitalization cannot be solely attributed to efgartigimod, and the current follow-up period is insufficient to ascertain the patient’s long-term prognosis. Future research, particularly multicenter randomized controlled trials, is essential to further investigate the efficacy, mechanisms of action, optimal dosing regimens, and long-term safety and effectiveness of efgartigimod in the treatment of anti-mGluR1 encephalitis.

## 4 Patient perspective

During discussions with the patient, it was explained that mGluR1 encephalitis is currently a relatively rare disease. An abnormal immune system attack on the brain can lead to symptoms such as dizziness and tremors, and may later be accompanied by sleep disorders and cognitive or behavioral changes. Some patients experience headache, weight loss, fatigue, nausea, or flu-like prodromal symptoms. A small subset of patients requires monitoring for potential tumor risks. According to multiple clinical case reports, approximately 40 per cent of patients showed significant improvement or complete remission following immunotherapy. Twenty percent of patients relapsed after discontinuing immunotherapy, but achieved complete remission or improvement upon resuming treatment. This indicates that for autoimmune-related diseases, the diagnostic and therapeutic processes are often prolonged, with symptoms potentially fluctuating during this period. The patient and their family have transitioned from a lack of understanding of the disease to providing positive feedback during monthly follow-ups. We initiated sequential immunotherapy with efgartigimod combined with low-dose rituximab. This approach maintained therapeutic efficacy while substantially reducing the risk of complications, thereby enhancing the safety profile. This combined therapeutic approach significantly alleviated symptoms and contributed to the long-term stabilization of the patient’s condition.

## Data Availability

The original contributions presented in the study are included in the article/supplementary material. Further inquiries can be directed to the corresponding author.
